# Regional, altitudinal, and age-specific heterogeneity of bronchiolitis hospitalization seasonality in Ecuador, 2007–2024, and implications for RSV immunoprophylaxis timing

**DOI:** 10.3389/fpubh.2026.1892098

**Published:** 2026-08-03

**Authors:** Jaime Angamarca-Iguago, Jaen Cagua-Ordóñez, Juan Marcos Parise-Vasco, Doménica de Mora, Mónica Escobar-Naranjo, Alfredo Bruno, Daniel Simancas-Racines

**Affiliations:** 1 Center for Evidence Ecosystems, Implementation Science and Decision-Making (CIDES), Facultad de Ciencias de la Salud y Bienestar Humano, Universidad Tecnológica Indoamérica, Ambato, Ecuador; 2 TEPHINET, Quito, Ecuador; 3 National Reference Laboratory for Influenza and Other Respiratory Viruses, INSPI, Guayaquil, Ecuador; 4 Dirección Nacional de Vigilancia Epidemiológica, Ministerio de Salud Pública del Ecuador, Quito, Ecuador; 5 Universidad Agraria del Ecuador, Guayaquil, Ecuador

**Keywords:** age-stratified surveillance, bronchiolitis, Ecuador, maternal RSV vaccine, nirsevimab, post-COVID rebound, respiratory syncytial virus, seasonality

## Abstract

**Background:**

Acute bronchiolitis, mainly attributed to respiratory syncytial virus (RSV), is the leading cause of infant hospitalization for lower respiratory tract disease worldwide. Nirsevimab and maternal RSVpreF (Abrysvo) confer about 6 months of protection, so timing relative to the season affects their impact. Ecuador spans the equator from sea level to valleys above 4,000 m and is considering introducing these products, yet lacks subnational evidence on the seasonality of bronchiolitis, altitudinal patterning, or post-pandemic stability.

**Methods:**

We conducted a national ecological time-series analysis of hospital discharges from the Ecuadorian Statistics Institute, 2007–2024 (19,788,097 discharges; 204 of 224 cantons had complete data and formed the analytic panel). The primary outcome was acute bronchiolitis (ICD-10 code J21) in children younger than 12 months (*n* = 23,190 nationally; 22,724 in the panel), with secondary outcomes across three RSV-relevant age bands, ICD-10 code J21 under 5 years, and two RSV-specific ICD-10 codes (J12.1 and J20.5; 2018–2024). K35 (appendicitis) was a negative control. Analyses combined circular seasonality metrics with block-bootstrap confidence intervals (CIs), year-by-year disaggregation, and complementary sensitivity analyses [Moving Epidemics Method (MEM), Bai–Perron breaks, and lagged climate correlations].

**Results:**

The weighted circular peak month of infant bronchiolitis hospitalizations shifted after the pandemic in Costa (+2.16 months), Sierra (+1.22), and Amazonía (+1.91), attenuating with altitude from +2.19 months below 500 m to +0.88 at 1,500–3,000 m. Costa was stable across 2022–2024; Sierra and, more so, Amazonía varied widely year to year (Amazonía peak ranged March–July, *n* = 37–42/year), so the pooled estimate should be read with caution. The composite RSV-coded outcome tracked ICD-10 code J21's timing closely in Costa (0.15-month difference) and Sierra (0.40), supporting ICD-10 code J21 as a reasonable RSV proxy in both, and the three regional peaks now span 2 months.

**Conclusion:**

Bronchiolitis seasonality in Ecuador changed after the pandemic, most clearly and stably in Costa; Sierra showed a transient 2022 perturbation with partial reversion by 2023–2024, and Amazonía's estimates remained unstable and underpowered. As region-tailored estimates are less stable in Sierra and Amazonía than in Costa, a single national window (nirsevimab February–April; maternal RSVpreF for pregnancies expected to deliver January–May) is a more defensible starting point, pending confirmation from further seasons and, ideally, laboratory-confirmed surveillance.

## 1 Introduction

Respiratory syncytial virus (RSV) is the leading global cause of acute lower respiratory tract infection (LRI) in young children and the most frequent identifiable cause of hospitalized bronchiolitis in infants (1). The Global Burden of Disease 2023 analysis estimated 2.50 million LRI deaths and 98.7 million disability-adjusted life-years lost worldwide in 2023, with children younger than 5 years carrying the highest absolute burden and an under-5 LRI mortality rate of 94.8 per 100,000 person-years (2). RSV-specific global modeling identifies RSV as a predominant viral cause of acute lower respiratory infection and of associated hospitalization in children younger than 5 years (3), consistent with earlier modeling for 2015 (4); the two antigenic subgroups, RSV A and RSV B, co-circulate worldwide with alternating or simultaneous predominance (5). Published case series of hospitalized infant bronchiolitis consistently attribute approximately 70% of cases in those younger than 12 months to RSV, with rhinovirus, human metapneumovirus, adenovirus, parainfluenza, and bocavirus accounting for the remainder (6–9).

Two RSV-targeted preventive interventions have rapidly changed how this disease can be prevented. Nirsevimab, a long-acting monoclonal antibody administered to all infants entering their first RSV season, demonstrated significant efficacy against medically attended RSV-associated lower respiratory tract infection in the pivotal phase 3 MELODY randomized trial of healthy late-preterm and term infants (10), with consistent efficacy across a pooled analysis of randomized trials (ClinicalTrials.gov, NCT03979313) (11). Real-world post-licensure cohorts in Catalonia subsequently reported 70%–90% effectiveness against RSV-associated bronchiolitis hospitalizations (12), demonstrating that universal immunoprophylaxis can rapidly reduce pediatric respiratory bed occupancy. In Spain's Region of Murcia, achieving 93.5% coverage under a continuous-training program resulted in an 80%–90% decline in RSV hospitalizations during the first implementation season (13), and the product was well-tolerated in 1,559 children followed by the Canadian National Vaccine Safety Network (14). The maternal RSV prefusion-F vaccine (RSVpreF, Abrysvo: Pfizer Inc., New York, USA), administered to pregnant individuals at 32–36 weeks of gestation, produces transplacental antibodies that protect newborns: in the pivotal Maternal Immunization Study for Safety and Efficacy (MATISSE) trial, it conferred significant efficacy against medically attended severe RSV lower respiratory tract illness in the first months of life (15). In the largest head-to-head comparison to date, a French population-based cohort of 164,140 infants in 2024–2025, nirsevimab at birth was associated with a 22% reduction in RSV-related lower-respiratory hospitalization at age 6 months compared with RSVpreF maternal vaccination, although vaccination at least 8 weeks before delivery achieved protection statistically indistinguishable from nirsevimab (16). Both products confer approximately 6 months of effective protection, making the timing of administration in relation to the local RSV epidemic decisive for their public health impact.

In high-latitude settings, RSV seasonality is sharply unimodal and winter-centered; current programmatic recommendations align administration with northern-hemisphere October–November or southern-hemisphere March–May windows (17). The 32-country pan-American RSV seasonality study by Couto and colleagues showed that the epidemic progresses from south to north, that the season in the Andes peaks earlier than in Central America, and that lower temperatures, decreased longitude, and elevation are independently associated with RSV circulation (17). A Costa Rican distributed-lag non-linear modeling study of respiratory hospitalizations across peripheral climate zones found that precipitation extremes, high humidity, and aerosol optical depth contributed more to respiratory hospitalizations than temperature, with effects varying substantially between Caribbean, Pacific, and border subregions (18). A systematic analysis of within-country RSV seasonality across China went further, defining discrete RSV transmission zones of provinces with shared circulation patterns, deriving zone-specific optimal immunization start months, and proposing that this zone-based framework is transferable to other countries (19). These regional precedents suggest that uniform national immunization timing is unlikely to be optimal across heterogeneous tropical and equatorial settings.

The coronavirus disease 2019 (COVID-19) pandemic produced an additional dimension of heterogeneity. Non-pharmaceutical interventions during 2020–2021 suppressed RSV circulation almost everywhere, and the post-pandemic rebound produced out-of-season peaks of varied magnitude, including off-season summer surges in temperate sentinel networks (20–22). The subsequent post-pandemic resurgence has been linked to immunity debt, immunological imprinting, and viral antigenic dynamics (23, 24), with national interrupted time-series analyses in France (25) and Korea (26) documenting both transient and sustained changes in pediatric respiratory care utilization. Latin America witnessed sparser post-pandemic surveillance: a hospital-based surveillance study in Arequipa, a high-altitude city in southern Peru, observed RSV detection rates rising from 0.8% in 2021 to 29.0% in 2023 with associations to relative humidity (27), and genotype-level RSV transmission has been characterized in Cali, Colombia, in the northern Andes (28). Pre-pandemic, laboratory-confirmed surveillance of outpatient acute respiratory infection in Ecuadorian children already found that RSV accounted for 15% of tested episodes and that RSV and influenza activity differed between the coast and the highlands (29), directly foreshadowing the regional heterogeneity we characterize here at national, hospitalization-based scale. Whether the equatorial Andean ecosystem of Ecuador experienced an analogous, regionally heterogeneous, post-COVID-19 shift in bronchiolitis seasonality has not been characterized.

Chile became, in 2024, the first Southern-Hemisphere country to implement a universal nirsevimab immunization program, achieving over 90% coverage and resulting in an estimated 80%–88% reduction in RSV-associated hospitalizations among infants; a counterfactual analysis attributed to the program reductions of approximately 25,620 outpatient attendances, 59,072 hospital bed-days, 25,632 intensive-care bed-days, and a net economic benefit of USD 23.5 million in 2024 (30). As neighboring Andean countries prepare similar programs, sub-national evidence on the actual timing and shape of RSV-related bronchiolitis hospitalization seasons is operationally critical.

Ecuador is a particularly informative natural laboratory. Spanning the equator from sea level to permanently inhabited settlements up to 4,000 m (with peaks exceeding 6,000 m) within approximately 300 km, the country comprises four distinct natural regions (Pacific coast, Andean highlands, Amazon basin and the Galápagos archipelago) with sharply different climatic regimes. Analyses of dengue and leptospirosis based on national hospital discharge data have highlighted strong spatial and climatic heterogeneity in arboviral and zoonotic transmission across these regions: a nationwide leptospirosis study using the INEC hospital discharge registry documented higher risk in tropical coastal and Amazonian cantons and intermittent peaks during the rainy seasons (31), and an age-stratified analysis of dengue across Ecuador's ecologically diverse provinces showed the progressive establishment of endemic transmission, expanding most recently into historically isolated coastal and Amazonian areas (32). Influenza phylogeographic work spanning the COVID-19 disruption also identified Ecuador as a center documenting changes in the global dispersal of seasonal influenza (22). By contrast, the seasonality and altitudinal distribution of childhood bronchiolitis hospitalizations in Ecuador have not been characterized, and the country is currently considering but has not yet implemented nirsevimab or the maternal RSVpreF vaccine.

We address three questions. First, does the seasonality of bronchiolitis hospitalizations in children younger than 12 months differ between Ecuador's natural regions and along its altitudinal gradient? Second, did the COVID-19 pandemic shift the timing or amplitude of these peaks, and, critically, is any such shift stable across individual post-pandemic years or dependent on a single season? Third, does a clinically defined outcome [bronchiolitis, International Statistical Classification of Diseases and Related Health Problems, 10th Revision (ICD-10) code J21] track the seasonality of RSV-specific coded diagnoses (ICD-10 codes J12.1 and J20.5) closely enough to support its use as a surveillance proxy, and how is the burden distributed across the age bands most relevant to nirsevimab, maternal RSVpreF vaccination and palivizumab (0– < 6, 6– < 12, and 12– < 24 months)? Because seasonality estimates–not RSV-specific efficacy data–are what this ecological design can supply, we treat the translation of our findings into a specific immunization-timing recommendation as a secondary, deliberately cautious objective rather than the primary contribution of the study; the answers are intended chiefly to characterize bronchiolitis seasonality in an equatorial Andean setting and to identify where the evidence is, and is not yet, solid enough to inform policy.

## 2 Materials and methods

### 2.1 Study design and data sources

This was a pre-specified national ecological time-series analysis of routinely collected hospital discharge data obtained from the national hospital discharge registry of the Ecuadorian National Institute of Statistics and Census (*Instituto Nacional de Estadística y Censos*, INEC) and covering 1 January 2007 to 31 December 2024 (33). Cantons were geo-referenced using the official INEC *División Político Administrativa* (DPA) cartography (34), population denominators were obtained from the official INEC projections (35), monthly canton-level climate variables came from a previously released national dataset (36), and the El Niño–Southern Oscillation (ENSO) Niño 1+2 sea-surface-temperature anomaly series was obtained from the National Oceanic and Atmospheric Administration (NOAA) Climate Prediction Center (37). Reporting follows the Strengthening the Reporting of OBservational studies in Epidemiology (STROBE) statement for observational studies (38) and the REporting of studies Conducted using Observational Routinely collected health Data (RECORD) extension for studies using routinely collected health data (39).

### 2.2 Cohort and outcomes

Age at admission is recorded by INEC using an age-unit code (*cod_edad*) whose numerical meaning changed between vintages of the discharge registry (2007–2014: 1 = days, 2 = months, 3 = years; 2015–2023: 1 = h, 2 = days, 3 = months, 4 = years; 2024: Spanish-language text labels). All age-derived variables in this study (the primary and secondary bronchiolitis outcomes, the RSV-specific outcomes, and the fine age bands described below) were derived using a vintage-aware decoding rule applied at the individual-record level before aggregation, so that a given numeric age-unit code is interpreted according to the coding scheme in force in the discharge year of that record. Canton of residence was similarly linked to the official DPA cantonal code using a vintage-aware rule (a two-digit intra-provincial index concatenated with the province code for 2007–2010; the four-digit DPA code directly for 2011–2024, with accent-normalized text-to-code matching for the 2024 Spanish-language export) and cross-checked against the official INEC cartography (34).

**Figure 1:**
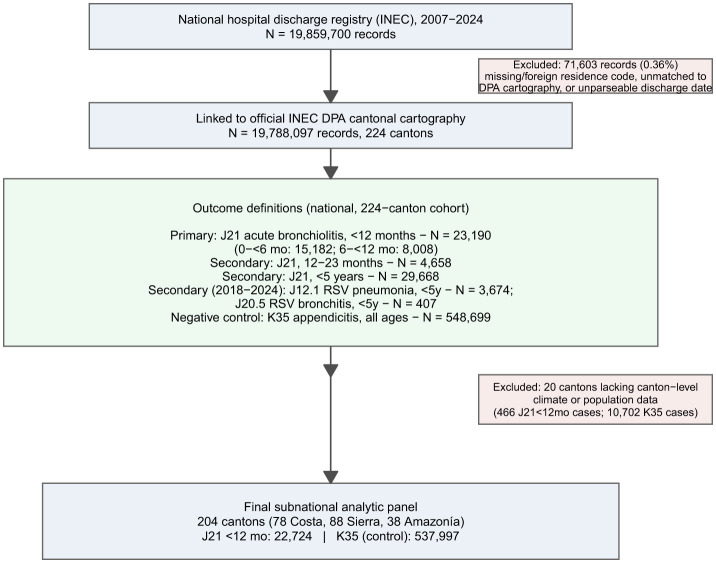
STROBE-style cohort flow diagram. The diagram outlines the selection criteria, exclusions, and final sample sizes of hospital discharge records, cantons, and cases for the primary analysis (ICD-10 code J21 in children younger than 12 months, with the 0– < 6, 6– < 12 and 12– < 24-month bands) and secondary/sensitivity analyses (ICD-10 code J21 in children younger than 5 years, ICD-10 codes J12.1 and J20.5 RSV-coded diagnoses individually and as a composite outcome, and the non-respiratory negative control K35 appendicitis) across Ecuador, 2007–2024.

After excluding records with missing or foreign residence codes and records that could not be linked to a valid canton under this rule (71,603 records, 0.36%, including a documented administrative-boundary recoding for the canton of La Concordia), the analysis included 19,788,097 hospital discharges linked to 224 cantons. The final analytic panel covered 204 cantons (78 Costa, 88 Sierra, and 38 Amazonía) with complete climate, population, and outcome data; cantons in the Galápagos archipelago (*n* = 3) and administratively disputed cantons in an undelimited inter-provincial border zone could not be matched to the climate dataset and are discussed as limitations. The cohort selection process, exclusions, and final sample sizes for the primary and sensitivity analyses are summarized in a STROBE-style flow diagram (Figure 1).

The primary outcome was acute bronchiolitis hospitalization (ICD-10 code J21) in children younger than 12 months (*n* = 23,190 nationally; 22,724 in the 204-canton analytic panel). Secondary outcomes were ICD-10 code J21 in three RSV-relevant age bands (0– < 6 months, 6– < 12 months, and 12– < 24 months), matching the indications for nirsevimab, maternal RSVpreF vaccination and optimized palivizumab use in infants and children younger than 2 years (10, 15); ICD-10 code J21 in children younger than 5 years (PAHO operational definition); and, restricted to 2018–2024, the period in which etiological coding of respiratory viruses stabilized at Ecuadorian hospitals, the RSV-specific ICD-10 codes J12.1 (RSV pneumonia) and ICD-10 code J20.5 (RSV bronchitis), used both individually and as a composite “RSV-coded” outcome (ICD-10 code J12.1 or J20.5). K35 (appendicitis) at all ages served as a non-respiratory, non-seasonal negative control throughout.

We chose ICD-10 code J21 in children younger than 12 months as the primary outcome because: (i) published evidence indicates that a substantial majority of hospitalized bronchiolitis in this age group is RSV-attributable, with laboratory-confirmed cohort estimates ranging from approximately 63 to 80% depending on setting and case mix (6–8, 40); (ii) this age range matches the indication for nirsevimab and the protective window of maternal Abrysvo (12, 16); and (iii) the ICD-10 code J21 is clinically robust to changes in diagnostic laboratory capacity over the study period. We explicitly acknowledge, however, that ICD-10 code J21 is a clinical rather than an etiological outcome, and that explicit RSV-coded diagnoses (ICD-10 codes J12.1 and J20.5) are themselves known to substantially under-capture true RSV burden in administrative hospital-discharge data relative to laboratory- or surveillance-based estimates, a pattern documented for RSV-coded diagnoses in adults (41) that is plausibly, although not directly, transferable to pediatric administrative coding. Accordingly, we did not treat the RSV-specific codes as a gold standard, but instead used them, in the restricted 2018–2024 window in which their coding is most stable, as an internal triangulation check on whether the seasonal timing captured by ICD-10 code J21 is consistent with that of the more specific (albeit far rarer) RSV-coded diagnoses (see Section 2.4.6).

### 2.3 Exposures and covariates

Each canton was classified into one of four natural regions (Costa, Sierra, Amazonía, and Galápagos) according to its province. Altitude (in m above sea level) for each canton was extracted by computing the centroid of the official INEC 2011 cantonal polygon and querying the Open Elevation application programming interface (API), derived from the Shuttle Radar Topography Mission (SRTM). We acknowledge that the centroid altitude is an approximation of the altitude of the cantonal head town, where hospital infrastructure is concentrated; for some large or topographically heterogeneous cantons, the discrepancy may exceed 1,000 m. Due to this approximation, altitude was analyzed only as a four-level categorical stratification (less than 500 m, 500–1,500, 1,500–3,000, and more than 3,000 m) rather than as a continuous variable; this altitudinal stratification is a pre-specified, primary analysis (Section 2.4.4), reported in parallel with, not subordinate to, the regional stratification.

Monthly climate variables at the canton level (land surface temperature, precipitation, NDVI) were obtained from a previously released dataset for Ecuador 2005–2024 derived from MODIS MOD11C3/MYD11C3, GPM IMERG V07B, and MODIS MOD13C2/MYD13C2 products (36). Monthly sea-surface-temperature anomalies for the Niño 1+2 region (the ENSO index most relevant to the Ecuadorian coast, encompassing 0–10°S and 90–80°W) were obtained from the NOAA Climate Prediction Center (37).

Population denominators (canton × year × age category, including an under-1-year and an under-5-year category) came from official INEC projections 2007–2035 (35).

### 2.4 Statistical analysis

The analytical unit was canton-month. Pre-specified analyses were performed within a triangulated framework, with circular seasonality metrics as the primary measure of phase change; the Moving Epidemics Method, Bai–Perron structural-break detection, and lagged climate correlations served as complementary, operational, and sensitivity analyses (detailed in Supplementary materials S1–S3) rather than as independent primary inference.

#### 2.4.1 Periods

Three analytic periods were pre-specified: pre-pandemic (2007–2019), pandemic (2020–2021, treated as a disruption interregnum and described separately), and post-pandemic (2022–2024). For simplicity, table and figure labels use “pre-COVID-19”/“post-COVID-19” interchangeably with “pre-pandemic”/“post-pandemic” to denote these same two periods.

#### 2.4.2 Moving epidemics method

For each region and period, we apply the Moving Epidemics Method (MEM) (42) to monthly aggregates of ICD-10 code J21 among children younger than 12 months, deriving the epidemic threshold and the start, peak, and end months of each season. We used the R package mem (version 2.18) with geometric-mean thresholds, a significance level of 0.95, and intensity thresholds at the 40th, 90th, and 97.5th percentiles.

#### 2.4.3 Circular seasonality metrics

For each region-period combination we computed: the weighted circular mean peak month (mapping calendar months to angles θ = 2π(*m* − 1)/12 weighted by mean monthly count), Pielou's evenness *J*′ = *H*/log(12) on the normalized 12-month distribution, the amplitude ratio (maximum monthly value divided by mean monthly value), and the signed phase shift bounded to [−6, +6] months between epochs (43). We pre-specified non-parametric block-bootstrap 95% confidence intervals (CIs) (2,000 monthly-block resamples) for between-period differences, following the framework applied by Couto et al. (17).

#### 2.4.4 Altitude-stratified circular metrics

Using the same weighted circular mean peak month, Pielou's evenness and amplitude ratio described above, we computed pre- vs. post-COVID-19 metrics for each of the four altitude strata, pooling across natural regions. This analysis directly addresses whether the altitudinal gradient in bronchiolitis seasonality implied by the country's topography is empirically detectable, independent of the regional stratification.

#### 2.4.5 Post-pandemic year-by-year stability

Because a 3-year post-pandemic window (2022–2024) is short relative to the multi-year re-stabilization of respiratory-virus seasonality documented elsewhere (44), we additionally computed the circular peak month, amplitude ratio, and Pielou's evenness separately for each of 2022, 2023, and 2024 within each natural region. This year-by-year disaggregation was pre-specified as the primary basis for judging whether the pooled 2022–2024 estimate for a given region should be treated as stable or provisional, pending further seasons of data.

#### 2.4.6 Etiological validation

To assess whether the primary, clinically defined outcome (ICD-10 code J21) tracks the seasonal timing of RSV-specific coded diagnoses, we restricted the panel to 2018–2024 (the window in which ICD-10 codes J12.1 and J20.5 are most stable) and computed the weighted circular peak month for ICD-10 code J21 (children younger than 5 years) and, separately, for the composite RSV-coded outcome (ICD-10 code J12.1 or J20.5, children younger than 5 years), by natural region. We treated a small absolute peak-month difference between the two outcomes as supportive of ICD-10 code J21's validity as an RSV seasonality proxy in that region, and treated regions with fewer than approximately 50 composite RSV-coded events over the 7-year window as underpowered for this comparison rather than as evidence against validity.

#### 2.4.7 Structural-change detection

To verify whether our pre-specified analytical periods align with objective changes in transmission regimes, we fitted Bai–Perron multiple-structural-break models (45) to the monthly time series of regional ICD-10 code J21 hospitalization rates per 100,000 infants using the R package strucchange (version 1.6-4). We modeled the time-series rate as a function of a constant intercept (rate ~ 1) to identify shifts in the baseline hospitalization level. The trimming parameter was set to *h* = 0.15, corresponding to a minimum segment duration of approximately 33 months (15% of the 216-month series) to ensure statistical stability. The optimal partition and the number of breakpoints *m* were selected by minimizing the Bayesian Information Criterion (BIC). The estimated break dates and their BICs were compared to the pre-specified period boundaries to verify alignment.

#### 2.4.8 Climate sensitivity

As an exploratory sensitivity analysis, we attempted to fit distributed lag non-linear models (DLNM) (46) to estimate potential non-linear lagged effects of temperature, precipitation, and the Niño 1+2 SST anomaly on monthly ICD-10 code J21 counts by region. Lag range was 0–3 months; lag-response was modeled with a natural cubic spline; the exposure-response with a natural cubic spline at quartile knots. However, due to a lack of robust model convergence under post-pandemic small-sample conditions (particularly in Amazonía and Sierra), we restricted the climate-sensitivity analysis to pre-specified Pearson and Spearman lagged correlation analyses (lags 0–3 months), which we present as descriptive context in line with the subregional framework of Chou-Chen and Parra-Rodríguez for tropical respiratory hospitalizations in Costa Rica (18).

Because both the climate exposures and the hospitalization rate are strongly autocorrelated monthly series sharing an annual seasonal cycle, ordinary Pearson significance testing on 216 monthly observations substantially overstates the number of effectively independent observations. We therefore report, alongside the nominal *p*-value, an autocorrelation-adjusted *p*-value derived from an effective sample size *N*_eff_ = *N*(1 − *r*_1*x*_*r*_1*y*_)/(1 + *r*_1*x*_*r*_1*y*_), where *r*_1*x*_ and *r*_1*y*_ are the lag-1 autocorrelations of the exposure and outcome series, respectively (47), and we apply the Benjamini–Hochberg false-discovery-rate (FDR) correction to the autocorrelation-adjusted *p*-values across all 3 × 4 × 4 = 48 region-by-exposure-by-lag tests performed. We regard only correlations surviving both corrections as robust; all others are reported as purely descriptive. We note that this lag-1 correction addresses short-range serial autocorrelation but does not fully remove the shared annual seasonal cycle common to both series; because both climate exposures and bronchiolitis hospitalization peak once per year, part of any surviving correlation, including the Costa precipitation association, may still reflect this shared seasonality rather than a lagged causal pathway, specifically, a limitation we return to in the Discussion.

#### 2.4.9 Negative control and diagnostic-effort adjustment

For all primary analyses we repeated the procedure with K35 (appendicitis) as the outcome to verify that detected seasonal signals were not artifacts of administrative or diagnostic-effort variation; the canton-month log of total hospital discharges (all causes) was retained in the analytic panel as a candidate diagnostic-effort covariate, although it was not ultimately required once the DLNM approach (which would have used it directly) was abandoned in favor of the descriptive correlation analysis described above.

### 2.5 Reproducibility and ethics

All analyses were performed in R 4.5.3 (R Foundation for Statistical Computing, Vienna, Austria) (packages: mem, circular, strucchange, dlnm, mgcv, and boot) and Python 3.9 (Python Software Foundation, Beaverton, OR, USA) (pandas and PyArrow). The aggregated canton-month panel and the analysis code are available from the corresponding author on reasonable request.

No submission to a Research Ethics Committee/Institutional Review Board (REC/IRB) was sought because the analysis used only publicly available, aggregated, de-identified INEC statistics and did not involve interaction with human participants, intervention, biological samples, or access to identifiable health records; no individual-level identifiable data were available to the investigators at any stage. This approach is consistent with Ecuadorian regulations governing Health-Research Ethics Committees (Ministry of Public Health standards for *Comités de Ética de Investigación en Seres Humanos*, CEISH) and with the Law of the National Statistical System, and follows the same rationale used in prior published analyses of this registry (36).

Regarding missing data, records in the national hospital discharge database with missing or foreign residence codes, or that could not be linked to a valid canton under the vintage-aware linkage rule described above (71,603 records, 0.36% of the raw database, including a documented administrative-boundary recoding for the canton of La Concordia), were excluded from cohort mapping (Figure 1). For subnational models, 20 cantons lacking population or climate data were excluded (including the three Galápagos cantons and administratively disputed cantons in an undelimited inter-provincial border zone), representing 466 infant ICD-10 code J21 cases and 10,702 K35 control cases nationally. No imputation was performed for missing meteorological (temperature, precipitation, NDVI) or population denominators; instead, analyses were restricted to canton-month observations with complete data. In R analysis scripts, missing values in the primary outcome fields were treated as zero only when the canton-month record was present in the national panel, but the count field was empty.

## 3 Results

### 3.1 Cohort characteristics

Across 18 calendar years (2007–2024) and 204 analyzable cantons (78 Costa, 88 Sierra, and 38 Amazonía) we identified 23,190 hospitalizations for acute bronchiolitis (ICD-10 code J21) in children younger than 12 months nationally (22,724 in the final subnational analytical panel after excluding 466 cases in 20 cantons lacking climate or population data; see Figure 1). Of the 23,190 national cases, 15,182 (65.5%) occurred in infants 0– < 6 months and 8,008 (34.5%) in infants 6– < 12 months; a further 4,658 hospitalizations occurred in children 12– < 24 months (all age-band figures reported here and in Table 1 use the national, 224-canton cohort, not the 204-canton analytic panel, since the fine age bands are a descriptive, not a climate/population-linked, analysis). In 2024 the estimated national under-one population, including cantons outside the analytic panel, was 266,123 infants: 150,237 (56.5%) in Costa, 99,633 (37.4%) in Sierra, 15,842 (5.95%) in Amazonía, and 411 (0.15%) in Galápagos. The Galápagos cantons are shown here for completeness only: as noted above, they are excluded from all analytic models because of missing canton-level climate data, alongside administratively disputed cantons in an undelimited inter-provincial border zone. The control outcome K35 (acute appendicitis) accumulated 548,699 hospitalizations across the panel (537,997 in the subnational panel after excluding 10,702 cases in the same 20 cantons; see Figure 1) and showed no seasonal pattern attributable to respiratory virus circulation.

**Table 1:** Fine age-band distribution of ICD-10 codes J12.1 and J20.5, and the composite RSV-coded outcome, Ecuador, 2018–2024 (national totals).

Outcome	0– < 6 months	6– < 12 months	12– < 24 months
ICD-10 code J21 (acute bronchiolitis)	6,585	3,336	1,807
ICD-10 code J12.1 (RSV pneumonia)	751	556	831
ICD-10 code J20.5 (RSV bronchitis)	74	46	84
ICD-10 codes J12.1 and J20.5 (composite RSV-coded)	825	602	915

### 3.2 Period-stratified circular seasonality metrics

Table 2 reports the weighted circular peak month, Pielou's evenness *J*′, and the amplitude ratio for each region by period, with 2,000-resample block-bootstrap 95% confidence intervals.

**Table 2:** Circular seasonality metrics for J21 (< 12 months) hospitalizations by region and period, Ecuador 2007–2024.

Region	Period	*n*	Peak month (95% CI)	Pielou's *J*^′^(95% CI)	Amplitude (95% CI)
Costa	Pre-COVID-19	9,888	3.87 (3.55–4.19)	0.972 (0.965–0.978)	1.74 (1.62–1.92)
Costa	Post-COVID-19	2,550	**6.03 (5.82–6.31)**	0.916 (0.892–0.932)	**2.71 (2.45–3.00)**
Sierra	Pre-COVID-19	6,081	2.84 (2.59–3.20)	0.963 (0.950–0.972)	1.73 (1.49–2.03)
Sierra	Post-COVID-19	2,257	4.06 (3.25–4.83)	0.959 (0.926–0.971)	1.74 (1.53–2.31)
Amazonía	Pre-COVID-19	618	3.42 (2.93–3.85)	0.972 (0.951–0.981)	1.73 (1.46–2.16)
Amazonía	Post-COVID-19	120	5.34 (3.87–6.92)	0.978 (0.921–0.983)	1.60 (1.45–2.60)

Point estimates with 95% block-bootstrap confidence intervals (2,000 resamples). Bold values indicate the Costa post-COVID-19 estimates (peak month and amplitude ratio), the largest and most statistically robust regional post-pandemic shift (non-overlapping 95% CIs).

**Figure 2:**
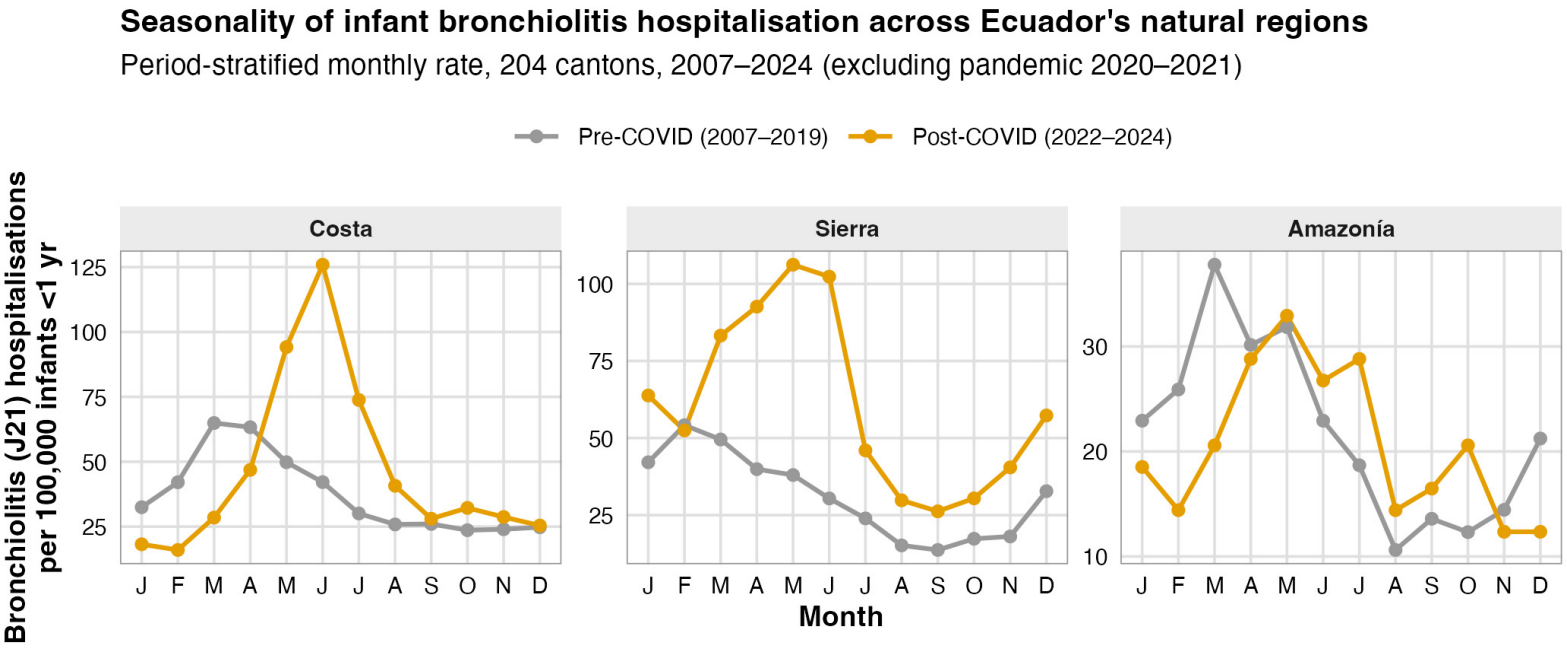
Seasonality of infant bronchiolitis hospitalization across Ecuador's natural regions. Period-stratified mean monthly hospitalization rate for acute bronchiolitis (ICD-10 code J21) in children younger than 12 months, expressed per 100,000 infants, by natural region, 2007–2024. Pre-COVID-19, 2007–2019; post-COVID-19, 2022–2024; the pandemic years 2020–2021 are excluded.

**Figure 3:**
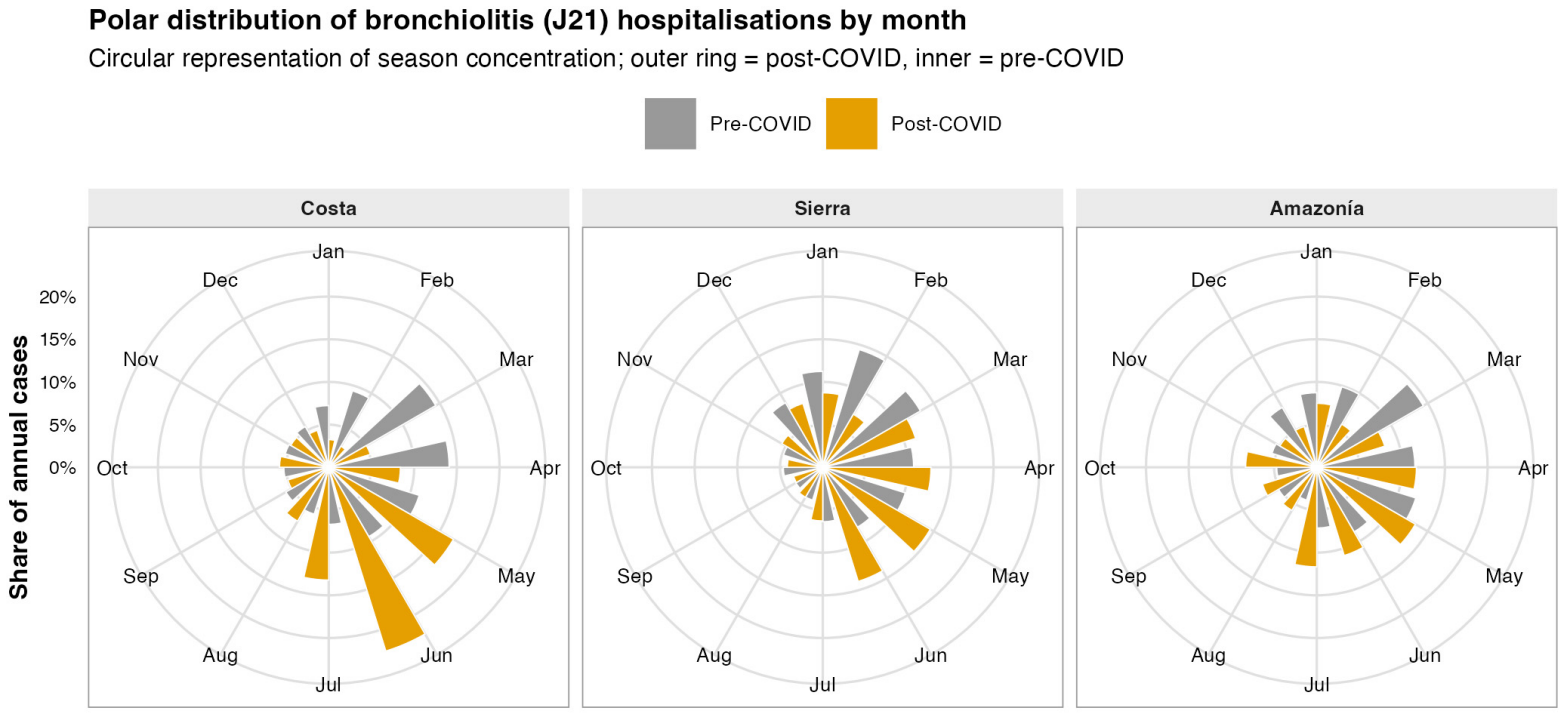
Polar distribution of monthly bronchiolitis (ICD-10 code J21) hospitalizations in children younger than 12 months by natural region, comparing the pre-COVID-19 (2007–2019) and post-COVID-19 (2022–2024) periods. Each bar shows the share of annual cases occurring in the corresponding calendar month.

Numerically, all three natural regions showed a post-pandemic phase shift in the same direction, although the strength and stability of this evidence differed markedly by region. Costa shifted by +2.16 months (3.87 → 6.03), with non-overlapping 95% confidence intervals and a 56% increase in seasonal amplitude (1.74 → 2.71, also non-overlapping). Given *n* = 9, 888 and *n* = 2, 550 pre- and post-pandemic events, respectively, this is the largest and best-supported of the three regional estimates. Sierra shifted by +1.22 months (2.84 → 4.06), with narrowly non-overlapping confidence intervals (upper pre-COVID-19 bound 3.20 vs. lower post-COVID-19 bound 3.25) and essentially unchanged amplitude (1.73 vs. 1.74). Amazonía shifted by +1.91 months (3.42 → 5.34). The post-pandemic estimate now rests on 120 events, up from 42 in an earlier, less complete build of this panel, and yields a materially narrower confidence interval than before. However, as detailed in Section 3.4, this pooled 3-year estimate masks considerable year-to-year instability and should still be interpreted cautiously (Figures 2, 3).

### 3.3 Altitude-stratified circular seasonality metrics

Table 3 reports the same metrics stratified by altitude band, pooling across natural regions, addressing whether the phase shift follows the country's altitudinal gradient independent of the regional classification.

**Table 3:** Circular seasonality metrics for ICD-10 code J21 (< 12 months) hospitalizations by altitude stratum and period, Ecuador 2007–2024.

Region	Period	*n*	Peak month (95% CI)	Amplitude (95% CI)	Pielou's *J*^′^(95% CI)
< 500 m	Pre-COVID-19	10,115	3.84 (3.51–4.16)	1.74 (1.61–1.93)	0.972 (0.966–0.978)
< 500 m	Post-COVID-19	2,584	6.03 (5.82–6.31)	2.69 (2.43–2.99)	0.917 (0.892–0.933)
500–1,500 m	Pre-COVID-19	351	4.05 (3.34–4.68)	1.64 (1.41–2.16)	0.976 (0.950–0.985)
500–1,500 m	Post-COVID-19	124	5.22 (4.15–6.34)	1.74 (1.55–2.70)	0.962 (0.903–0.973)
1,500–3,000 m	Pre-COVID-19	1,800	3.38 (3.00–3.73)	1.75 (1.50–2.06)	0.974 (0.961–0.982)
1,500–3,000 m	Post-COVID-19	523	4.26 (3.79–4.81)	1.90 (1.61–2.37)	0.960 (0.930–0.973)
>3,000 m	Pre-COVID-19	4,321	2.68 (2.40–3.09)	1.83 (1.51–2.26)	0.954 (0.936–0.967)
>3,000 m	Post-COVID-19	1,696	3.97 (2.97–4.95)	1.73 (1.57–2.55)	0.956 (0.906–0.969)

Point estimates with 95% block-bootstrap confidence intervals (2,000 resamples).

The phase shift was largest in the coastal lowlands below 500 m (+2.19 months, non-overlapping confidence intervals, *n* = 10, 115 pre- and 2, 584 post-pandemic events) and smallest in the intermediate 1,500–3,000 m band (+0.88 months, intervals also non-overlapping). The high-altitude stratum above 3,000 m, dominated by Sierra cantons, showed an intermediate shift (+1.29 months, overlapping intervals: pre-COVID-19 upper bound 3.09 vs. post-COVID-19 lower bound 2.97) with essentially unchanged amplitude, mirroring the regional Sierra result; this overlap means the >3,000 m shift should be regarded as less certain than the below-500 and 1,500–3,000 m estimates. The 500–1,500 m band had the smallest sample in both periods (351 and 124 events), the widest confidence intervals of the four strata, and its estimate should be treated as the least precise. This altitude-stratified analysis, requested to substantiate the altitudinal component of our study title and objectives, corroborates the regional pattern: the post-pandemic shift attenuates with increasing altitude, consistent with a lowland-centered perturbation rather than a uniform national phenomenon.

### 3.4 Post-pandemic year-by-year stability

Because the post-pandemic window spans only 3 years, we disaggregated the circular metrics for each region by individual year (Table 4) to assess whether the pooled 2022–2024 estimates in Section 3.2 reflect a stable pattern or are driven by a single anomalous year.

**Table 4:** Year-by-year circular seasonality metrics for ICD-10 code J21 (< 12 months) hospitalizations, post-pandemic period, by region.

Region	Year	*n*	Peak month (95% CI)	Amplitude (95% CI)	Pielou's *J*^′^(95% CI)
Costa	2022	784	6.28 (5.99–6.71)	3.43 (3.11–3.80)	0.846 (0.810–0.875)
Costa	2023	1,000	5.74 (5.49–6.20)	2.35 (2.15–2.87)	0.935 (0.912–0.951)
Costa	2024	766	6.05 (5.78–6.47)	2.43 (2.02–2.80)	0.936 (0.913–0.951)
Sierra	2022	724	5.27 (4.71–5.59)	2.90 (2.62–3.51)	0.866 (0.821–0.898)
Sierra	2023	843	3.15 (2.52–4.28)	1.77 (1.66–2.18)	0.969 (0.945–0.973)
Sierra	2024	690	3.30 (2.86–3.99)	1.69 (1.54–2.42)	0.953 (0.920–0.976)
Amazonía	2022	41	5.27 (4.48–6.07)	3.22 (2.27–5.00)	0.879 (0.673–0.898)
Amazonía	2023	37	3.63 (1.11–10.71)	2.59 (1.82–4.29)	0.921 (0.732–0.922)
Amazonía	2024	42	7.05 (1.02–11.00)	2.86 (1.85–4.70)	0.931 (0.722–0.955)

Point estimates with 95% block-bootstrap confidence intervals (2,000 resamples).

Costa's peak month was stable across all three post-pandemic years (late May to late June) with tightly overlapping confidence intervals, supporting the pooled estimate in Table 2 as a genuine, reproducible feature rather than an artifact of any single year. Sierra showed an atypical 2022 (peak month 5.27, late May) that reverted toward its pre-pandemic timing by 2023–2024 (peak months 3.15 and 3.30, mid-March). This suggests that 2022, the year of school reopening and non-pharmaceutical interventions (NPI) withdrawal, carried a transient, immunity-debt-type perturbation that had substantially resolved by 2023, so the pooled Sierra estimate in Table 2 partly reflects this transient signal. Amazonía showed no consistent year-to-year pattern at all: the peak month ranged from March (2023) to July (2024), with only 37–42 events per year, and the individual-year confidence intervals are correspondingly enormous (2023: 1.11–10.71; 2024: 1.02–11.00, each spanning the full calendar essentially). The pooled 3-year Amazonía estimate in Table 2 has a numerically narrower bootstrap interval than in an earlier, less complete build of this analysis, but this year-by-year breakdown, and in particular the near-uninformative individual-year intervals reported here, shows directly that the pooled interval's apparent precision reflects the pooling procedure rather than a stable underlying seasonal signal. The Amazonía post-pandemic estimate should still be regarded as provisional pending further post-pandemic seasons.

### 3.5 Complementary and sensitivity analyses

Three further analyses were conducted as complementary, operational, or sensitivity checks rather than as independent primary inference; full results are reported in Supplementary materials S1–S3.

The Moving Epidemics Method (42), applied to the monthly ICD-10 code J21 series, provided a convergent post-pandemic operational threshold in Costa, but convergence was incomplete in four of the remaining five region-period strata (Costa pre-pandemic, both Sierra periods, and Amazonía post-pandemic); region-by-period thresholds and convergence status are reported in Supplementary Table S1. As convergence was incomplete across strata, the Moving Epidemics Method (MEM) was retained only as an operational cross-check, to be read alongside, not in place of, the circular metrics in Section 3.2.

Bai–Perron structural-break models (rate ~ 1), fitted to verify whether our pre-specified periods align with objective changes in transmission regimes, test for shifts in the *level* of the monthly hospitalization rate; as specified in the Methods (Section 2.4.7), this model cannot directly test a shift in the within-year *timing* of the seasonal peak. The optimal partitions placed breakpoints in Costa and Sierra close to the 2022 school-reopening/NPI-withdrawal period, which we interpret as corroborating, not validating, a concurrent phase change; in Amazonía, the optimal partition did not select a 2022 breakpoint, reinforcing that the Amazonía finding should be treated as provisional (full breakpoint dates and BIC values in Supplementary Table S2).

**Figure 4:**
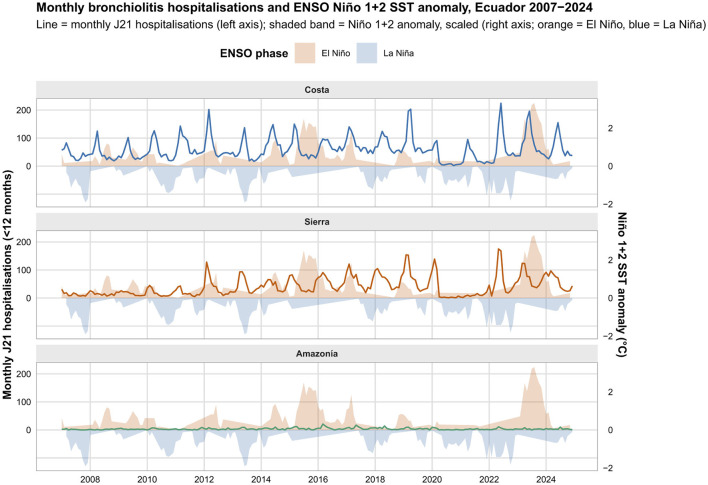
Descriptive time-series overlay of monthly bronchiolitis (J21) hospitalizations in children younger than 12 months (solid line, left axis) and the raw ENSO Niño 1+2 sea-surface-temperature anomaly (shaded band, right axis; orange, El Niño; blue, La Niña), Ecuador 2007–2024. The two series are plotted on independent axes: the left axis reports only non-negative hospitalization counts, and the right axis reports the anomaly in °C. This is a descriptive exposure-outcome visualization over time, not a fitted statistical model.

**Figure 5:**
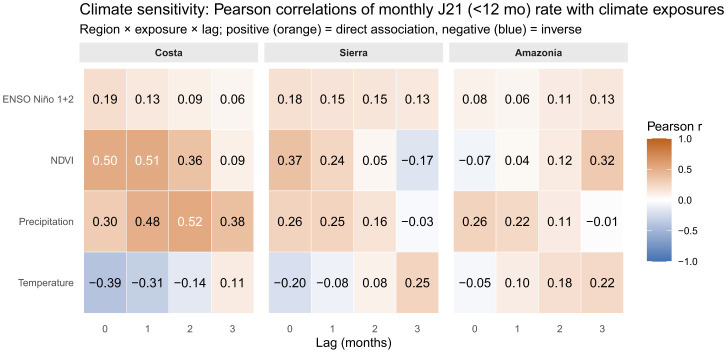
Lagged Pearson correlation coefficients (*r*) between monthly climate exposures (temperature, precipitation, NDVI, and the ENSO Niño 1+2 anomaly) and the monthly ICD-10 code J21 hospitalization rate in children younger than 12 months, by natural region (Costa, Sierra, and Amazonía) and lag (0–3 months) for the study period 2007–2024, based on the corrected analytic panel. The nominal sample sizes are *N* = 216 months at lag 0, *N* = 215 at lag 1, *N* = 214 at lag 2, and *N* = 213 at lag 3; every cell-specific Pearson correlation coefficient is annotated regardless of statistical significance. As both series are strongly autocorrelated, nominal *N* overstates the number of effectively independent observations; Sections 2.4.8 and 3.5 report autocorrelation-adjusted, Benjamini–Hochberg FDR-corrected significance for each cell, and only a subset of correlations (notably Costa precipitation at all four lags) survive both corrections. Shading indicates the strength and direction of the raw correlation (orange for positive, blue for negative); it does not by itself indicate which correlations are robust to the adjustments described in the main text.

Lagged correlations between regional climate exposures and the ICD-10 code J21 hospitalization rate were tested using an autocorrelation-adjusted effective sample size and a Benjamini–Hochberg FDR correction across all 48 region-by-exposure-by-lag tests (Section 2.4.8). Costa precipitation was the most consistent survivor, significant at all four lags (*p*_FDR_ ≤ 0.046); a handful of other region-exposure combinations survived at one to three lags, and ENSO Niño 1+2 anomalies did not survive correction in any region (full lag-by-lag results are shown in Supplementary Table S3; time-series overlay is shown in Figure 4, correlation summary is depicted in Figure 5). As both series share strong annual autocorrelation that the lag-1 adjustment does not fully remove, even the surviving correlations should be read as robust associations rather than as evidence that fully excludes shared seasonality as an explanation.

### 3.6 Etiological validation: ICD-10 code J21 vs. RSV-specific coded diagnoses

Restricting the panel to 2018–2024, the period in which coding of RSV-specific diagnoses (ICD-10 codes J12.1 and J20.5) is most stable, we compared the circular peak month of the all-cause primary outcome (ICD-10 code J21, children younger than 5 years) with that of the composite RSV-coded outcome (ICD-10 code J12.1 or J20.5, children younger than 5 years) within each region (Table 5).

**Table 5:** Etiological validation: peak month of ICD-10 code J21 vs. the composite RSV-coded outcome (ICD-10 codes J12.1 and J20.5), children younger than 5 years, 2018–2024.

Region	Outcome	*n*	Peak month (95% CI)
Costa	ICD-10 code J21 (all-cause)	6,379	5.03 (4.61–5.38)
Costa	ICD-10 codes J12.1 and J20.5 (RSV-coded)	1,749	4.88 (4.43–5.25)
Sierra	ICD-10 code J21 (all-cause)	5,404	2.98 (2.49–3.74)
Sierra	ICD-10 codes J12.1 and J20.5 (RSV-coded)	1,887	2.58 (2.15–3.79)
Amazonía	ICD-10 code J21 (all-cause)	356	3.51 (2.57–4.50)
Amazonía	ICD-10 codes J12.1 and J20.5 (RSV-coded)	18	5.40 (2.21–6.23)

Point estimates with 95% block-bootstrap confidence intervals (2,000 resamples).

In Costa, where the composite RSV-coded outcome accumulated 1,749 events over the 7-year window, its peak month (4.88, 95% CI 4.43–5.25) differed from that of ICD-10 code J21 (5.03, 4.61–5.38) by only 0.15 months, with substantially overlapping confidence intervals. In Sierra (1,887 RSV-coded events), the corresponding difference was 0.40 months [2.58 (2.15–3.79) vs. 2.98 (2.49–3.74)], again with overlapping intervals. Both differences are well within the resolution of a monthly circular metric, and the confidence-interval overlap provides formal, not just descriptive, support for using ICD-10 code J21 as a reasonable clinical proxy for RSV seasonality in these two regions, where the great majority of the pediatric population resides. In Amazonía the composite RSV-coded outcome accumulated only 18 events over 7 years, an order of magnitude too few to estimate a seasonal peak reliably [peak month 5.40, 95% CI 2.21–6.23, vs. 3.51 (2.57–4.50) for ICD-10 code J21]; although these two intervals happen to overlap, the RSV-coded interval spans nearly half the calendar and is uninformative on its own, so we regard this comparison as underpowered rather than as evidence either for or against ICD-10 code J21's validity in Amazonía, and note it as a specific priority for local etiological surveillance. Nationally, across the 2018–2024 restricted window, ICD-10 code J21 accumulated 6,585, 3,336, and 1,807 hospitalizations in the 0– < 6, 6– < 12, and 12– < 24 month bands respectively, while the composite RSV-coded outcome accumulated 825, 602, and 915 in the same bands (Table 1); the relatively larger contribution of RSV-coded diagnoses in the 12– < 24-month band likely reflects, at least in part, the lower prior probability of RSV testing/coding in the youngest, most clinically obvious bronchiolitis cases rather than a true shift in the underlying age distribution of RSV disease.

### 3.7 Policy translation

**Figure 6:**
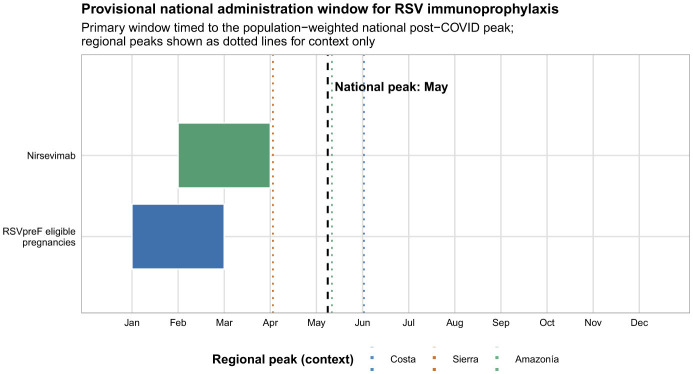
Provisional national administration window for nirsevimab and maternal RSVpreF (Abrysvo), derived from the population-weighted national post-COVID-19 (2022–2024) circular peak month of bronchiolitis hospitalization, with the three regional peak months shown for context. Dashed lines mark the national and regional peak months. For RSVpreF, the bar represents the approximate operational vaccination period among eligible pregnancies; eligibility should be determined by 32–36 weeks' gestation and an expected delivery date during January–May, not by a blanket calendar month of vaccination.

Table 6 and Figure 6 translates the post-pandemic peak month into an administration window under a single national policy, which we adopt as the primary framing given the year-by-year instability documented in Section 3.4. The population-weighted national post-pandemic peak month, weighted by the 2024 under-one population of Costa, Sierra, and Amazonía, was May (circular estimate 5.2). We emphasize what this number can and cannot support. A region-tailored schedule, by construction, matches each region's estimated peak exactly (0-month misalignment), so any single national window on either coverage or timing-proximity metrics cannot statistically outperform it. We do not, therefore, present the fact that the national window falls within 2 months of every region's peak (Costa peak June, 1 month from the national peak; Sierra peak April, 1 month from the national peak; and Amazonía peak May, coincident with the national peak) as evidence favoring the national option over the region-tailored one, since that comparison is close to tautological once a tolerance is chosen after seeing how tightly the regional peaks already cluster. The substantive, non-circular finding is the clustering itself: under the corrected panel, the three regional estimates now differ from the national peak by at most 1 month each, a materially smaller spread than in an earlier, less complete build of this analysis. This instability, documented for Sierra and, especially, Amazonía (Section 3.4), undermines the evidentiary basis for a region-tailored schedule specifically and independently of any coverage arithmetic. Combined with the operational simplicity of a single administration window, we consider the single national window the more defensible default at this time. We report the region-specific peaks in Table 6 for completeness and as a secondary, hypothesis-generating basis for future refinement, not as a coverage comparison; a region-tailored schedule is, in our view, premature given the instability documented in Section 3.4, rather than statistically disfavored by the national window's coverage.

**Table 6:** Provisional national administration window for nirsevimab and maternal RSVpreF (Abrysvo), derived from the population-weighted national post-COVID-19 (2022–2024) circular peak month (May), with region-specific peaks shown for context only.

Schedule component	National	Costa (context)	Sierra/Amazonía (context)
Post-COVID-19 peak	May	June	April/May
Nirsevimab window (birth cohort)	**February–April**	March–May	January–March/February–April
RSVpreF eligibility^*^ (32–36-week gestation)	**Expected delivery Jan–May**	Expected delivery Feb–Jun	Expected delivery Dec–Apr/Jan–May

^*^Not a fixed vaccination calendar: pregnant individuals are eligible once they reach 32–36 weeks of gestation, provided their expected delivery date falls within the window shown, so that antibody transfer precedes the infant's first RSV season (see Policy Translation [Section 3.7] above). Bold values indicate the recommended single national administration window.

The nirsevimab window in Table 6 is a direct calendar recommendation: because nirsevimab is administered to the infant at or shortly after birth, an administration window timed ahead of the national peak (February–April) is operationally equivalent to a birth-cohort recommendation. The maternal RSVpreF window requires an additional translation step that the calendar window alone does not convey. RSVpreF is indicated at 32–36 weeks of gestation (15), so the relevant operational criterion is not the calendar month in which vaccination occurs but whether a given pregnancy's expected delivery date falls before or during the national RSV window: in practice, this means offering RSVpreF to pregnant individuals who reach 32–36 weeks of gestation with an expected delivery date from approximately January through the peak month (May), so that transplacental antibody transfer precedes the infant's first RSV season, rather than vaccinating all pregnant individuals in a fixed calendar window irrespective of expected delivery date. Antenatal care schedules would need to explicitly flag eligibility for the expected delivery date, rather than applying the January–March window as a blanket vaccination period.

## 4 Discussion

In this national analysis of 23,190 infant bronchiolitis hospitalizations spanning 18 years and 204 cantons, the seasonal timing of bronchiolitis hospitalization in children younger than 12 months shifted after the COVID-19 pandemic in all three of Ecuador's natural regions and along its altitudinal gradient, most consistently and most strongly in the coastal lowlands. We deliberately frame this as a study of bronchiolitis hospitalization seasonality with cautious, secondary implications for RSV immunoprophylaxis policy, rather than as a policy-prescriptive RSV timing study: the ecological, hospital-discharge design we use can characterize when clinical bronchiolitis peaks, but cannot on its own establish either the RSV-attributable fraction driving that peak or the long-term stability of the post-pandemic pattern, both of which bear directly on how firmly any timing recommendation should be stated.

The best-supported finding is the coastal (Costa) phase shift: 3.87 (95% CI 3.55–4.19) to 6.03 (5.82–6.31), a +2.16-month shift with non-overlapping confidence intervals, a 56% increase in seasonal amplitude, and a peak month that was stable across all three individual post-pandemic years (2022, 2023, and 2024; Table 4). This finding is coherent with the pan-American characterization by Couto et al. (17), who reported that RSV epidemics progress from south to north across the Americas and that lower temperatures, decreased longitude and elevation are independently associated with RSV circulation; with the post-pandemic resurgence of RSV and influenza documented in the high-altitude Andean city of Arequipa, Peru (27); and with the broader international literature documenting delayed, reorganized or amplified post-pandemic RSV seasonality (20, 21, 44, 48–50). Sentinel laboratory-based surveillance within Ecuador has similarly indicated delayed RSV season onset after the pandemic (D. De Mora et al., poster presented at the 13th International RSV Symposium, Foz do Iguaçu, Brazil, 12–15 March 2025); the present canton-census analysis is consistent with that signal.

By contrast, the Sierra and Amazonía post-pandemic estimates require considerably more caution, and our year-by-year disaggregation (Section 3.4) is, in our view, as important a finding as the pooled phase-shift estimates themselves. Sierra's pooled post-pandemic peak (4.06) was driven substantially by an atypical 2022 (peak month 5.27) that had largely reverted toward the pre-pandemic timing by 2023–2024 (peak months 3.15 and 3.30); this pattern is compatible with a transient immunity-debt-type perturbation coinciding with school reopening and NPI withdrawal in Ecuador in mid-2022, superimposed on an otherwise comparatively stable Andean seasonal cycle, rather than a durable shift of the same character as Costa's. Amazonía's pooled estimate, although numerically more precise than in an earlier build of this analysis owing to a larger recovered sample (120 vs. 42 post-pandemic events), showed no consistent year-to-year pattern at all (peak month ranging from March to July across 2022–2024, with only 37–42 events per year); the Bai–Perron structural-break analysis (Section 3.5) reinforces this caution, since the BIC-optimal partition for Amazonía did not select a 2022 breakpoint at all, unlike Costa and Sierra. We interpret the Amazonía post-pandemic finding as hypothesis-generating rather than as an established, stable feature of that region's current seasonality.

The altitude-stratified analysis (Section 3.3, Table 3), added to substantiate the altitudinal component of our study objectives, showed a phase shift that attenuated with increasing altitude (from +2.19 months below 500 m to +0.88–+1.29 months above 1,500 m), broadly paralleling the regional pattern and consistent with a lowland-centered post-pandemic perturbation rather than a uniform national phenomenon. In Ecuador, stringent non-pharmaceutical interventions (NPIs) were implemented from March 2020 (national state of emergency, mobility curfews, mandatory masking, suspension of in-person schooling), with a phased resumption beginning in mid-2021, culminating in a mandatory nationwide return to face-to-face classes and the lifting of outdoor masking by May 2022. Ecuador's distinct regional school calendars (Sierra September–June; Costa May–January) meant that this resumption coincided with different phases of the local transmission cycle and climatic seasons; in the coastal lowlands, the resumption of childhood contact networks in May 2022 coincided with the onset of the dry season and, in 2023, a strong El Niño event (Niño 1+2 anomaly +3.30 °C in August 2023). This timing is compatible with a hydroclimatic contribution to the coastal shift. However, the ecological design cannot establish one, consistent with analyses linking climate variability to respiratory virus seasonality elsewhere (18, 51, 52). We note, however, that Costa's post-pandemic seasonal amplitude was in fact highest in 2022 (3.43; Table 4), the year of school reopening and NPI withdrawal but not the year of the strongest El Niño anomaly (2023, amplitude 2.35), and that peak timing was similarly consistent across both El Niño and non-El Niño post-pandemic years; taken together with the year-by-year results in Section 3.4, this pattern is more consistent with NPI withdrawal/immunity debt as the dominant driver of the coastal shift, with the 2023 El Niño event, and the coastal precipitation association more generally, plausibly contributing to or modulating its magnitude rather than driving its onset. With the corrected panel, the lagged coastal precipitation correlation is now stronger than we previously estimated (Pearson *r* = 0.52 at a 2-month lag, vs. *r* = 0.33 in an earlier, less complete build of this analysis), which we regard as a more reliable estimate of this association given the larger, more complete underlying sample; unlike the ENSO correlations, which did not survive correction in any region, the Costa precipitation correlation remains significant at all four lags after both an autocorrelation-adjusted effective sample size and Benjamini–Hochberg correction for the 48 tests performed (Section 3.5). It is the most consistent, all-lag survivor among the handful of exposure–region–lag combinations that do survive both corrections, which also include Costa temperature and NDVI, Sierra NDVI, and, more modestly, several Amazonía exposures (Section 3.5). This increases our confidence in the coastal precipitation association specifically, while reinforcing that the great majority of the other, unadjusted correlations we report should be read as descriptive rather than confirmatory. The caveat about causal attribution nonetheless stands regardless of statistical robustness, since an ecological design cannot distinguish a direct hydroclimatic effect from confounding by unmeasured, co-varying seasonal factors.

The etiological-validation analysis (Section 3.6) provides reassurance, in Costa and Sierra specifically, that ICD-10 code J21 is a reasonable proxy for RSV seasonality: the composite RSV-specific outcome (ICD-10 codes J12.1 and J20.5) peaked within 0.15 and 0.40 months of ICD-10 code J21 in these two regions, respectively, despite RSV-specific ICD-10 coding being, as documented in adult administrative data, prone to substantially under-capturing true RSV burden relative to laboratory-based surveillance (41). This agreement, in the two regions where the great majority of Ecuador's under-one population resides, is the strongest evidence we can offer from an administrative dataset that the clinical bronchiolitis signal we analyze is not badly misaligned with RSV circulation specifically; it does not, and cannot, substitute for prospective laboratory-confirmed surveillance, which remains a priority, particularly for Amazonía where the RSV-coded sample (18 events over 7 years) was too small to evaluate. The fine age-band results (Table 1) show that the great majority of ICD-10 code J21 hospitalizations (65.5%) occur in infants younger than 6 months, the population in which nirsevimab and maternal RSVpreF are expected to have their largest absolute impact, while RSV-coded diagnoses were comparatively more evenly distributed across the 0– < 24-month range, a pattern plausibly related to differential testing/coding practice by age rather than to the true age distribution of RSV disease, and worth confirming with etiological data.

Taken together, these findings lead us to a more conservative policy translation than a strict region-tailored schedule would imply. Nirsevimab and the maternal RSV prefusion-F vaccine each confer approximately 6 months of protection (10, 15), and real-world experience from Chile's 2024 universal nirsevimab program (30), Catalonia and the Region of Murcia in Spain (12, 13). Canadian post-licensure surveillance (14) demonstrates that well-timed, high-coverage immunoprophylaxis can substantially reduce RSV-associated hospitalization. However, because (i) the regional peaks under the corrected panel now cluster within a 2-month window (Costa June, Amazonía May, Sierra April), so that a region-tailored schedule would differ from a single national window by at most 1 month per region, and (ii) the post-pandemic estimates that would underpin a region-tailored schedule for Sierra and, especially, Amazonía are demonstrably unstable across individual post-pandemic years (Section 3.4), we consider a single national window (nirsevimab February–April; maternal RSVpreF eligibility for pregnant individuals at 32–36 weeks' gestation whose expected delivery date falls approximately January–May) the more defensible starting point for Ecuadorian policy at this time. We deliberately do not rest this recommendation on the fact that the national window falls within a 2-month tolerance of every regional peak (Table 6): because a region-tailored schedule matches each region's peak exactly by construction, it cannot be outperformed by a national window on that metric, and presenting an author-chosen tolerance as if it demonstrated a population-health benefit would repeat, in a different form, the overclaiming the original review correctly identified. The regional and altitudinal heterogeneity we document is retained as a secondary, monitoring-relevant finding rather than as the basis for an immediately implementable region-tailored schedule. This is a more cautious translation than the transmission-zone framework developed for China (19), reflecting both the shorter, less stable post-pandemic observation window available in Ecuador and the smaller share of the population residing in the region (Amazonía), which has the most divergent and least stable estimated timing.

From a surveillance perspective, the Moving Epidemics Method (MEM) offers operational advantages over traditional endemic channels (*canales endémicos*), which rely on static, calendar-month percentile thresholds; MEM instead models the epidemic curve as a whole, separating pre-epidemic, epidemic, and post-epidemic phases via dynamic thresholding, a distinction of practical relevance for timing short-lived immunoprophylaxis products.

The strengths of this study include the use of 18 years of national, person-level hospital discharge data covering 204 cantons; a vintage-aware harmonization of the age-at-admission and canton-of-residence coding fields, whose meaning changed between INEC discharge-registry vintages, applied uniformly across the study period; the use of a non-respiratory negative control (K35, acute appendicitis); an explicit, pre-specified etiological-validation triangulation against RSV-specific coded diagnoses; pre-specified altitude-stratified and year-by-year post-pandemic analyses; and the triangulated analytical framework combining the Moving Epidemics Method (42), circular seasonality statistics (43) and structural-break detection. Several limitations must be acknowledged. ICD-10 code J21 remains a clinical, not an etiological, outcome. Our etiological-validation analysis supports its use as an RSV proxy in Costa and Sierra, but this could not be confirmed in Amazonía, and the true RSV-attributable fraction has not been directly quantified for Ecuador with laboratory-confirmed data. Non-RSV respiratory pathogens (human metapneumovirus, influenza, parainfluenza, rhinoviruses) also cause bronchiolitis and may exhibit distinct seasonal peaks; if their contribution varies importantly by region or altitude, our seasonality estimates could partly reflect non-RSV circulation, particularly outside Costa and Sierra.

Geographically, the Galápagos archipelago and administratively disputed cantons were excluded due to a lack of canton-level climate data, so the findings do not generalize to insular Ecuador. Cantonal altitude was approximated from polygon centroids rather than population-weighted or head-town elevations, which is why we restricted altitude to a categorical stratification; human settlements in Ecuador are in any case confined to altitudes below approximately 4,100 m despite topographic peaks exceeding 6,000 m, so our altitudinal analysis reflects exposure within permanently inhabited valleys and slopes. Hospitalization also captures only severe disease reaching the inpatient setting. Hospital-discharge data conflate the seasonal signal with the altitudinal and rural–urban gradient in access to care: a finer provincial stratification within Sierra could partly reflect when families can reach a hospital rather than when the virus circulates, and our three-region aggregation may mask within-Sierra variation.

Most importantly for the policy translation, the post-pandemic observation window remains short (3 years). Several years are typically required before respiratory-virus seasonal patterns fully re-stabilize after a pandemic-scale disruption (44), and our own year-by-year analysis (Section 3.4) demonstrates directly that the Sierra and Amazonía pooled estimates are not yet stable. We regard this instability, rather than sample size alone, as the central reason for favoring a single national policy over a region-tailored one at this time.

Two further limitations concern our statistical methods. The autocorrelation adjustment applied to the climate-sensitivity correlations (Section 2.4.8) corrects for lag-1 serial dependence but not for the shared annual seasonal cycle common to both climate exposures and hospitalization counts, so the Costa precipitation association that survives correction should be interpreted as a robust *association* rather than as evidence that has fully excluded shared seasonality as an explanation. Secular improvements in diagnostic coding over the study period also preclude inference from absolute case counts; we therefore restricted inference to relative seasonal metrics, which are less sensitive to time-varying diagnostic effort, and analyzed the RSV-specific codes (ICD-10 codes J12.1 and J20.5) only from 2018 onward for the same reason.

In conclusion, bronchiolitis hospitalization seasonality in Ecuador changed after the COVID-19 pandemic, with the clearest and most stable shift in the coastal lowlands (Costa); Sierra's post-pandemic estimate was partly driven by a transient 2022 perturbation that had largely reverted by 2023–2024, and Amazonía's post-pandemic estimates remained unstable and underpowered across individual years. This certainty gradient is corroborated, where sample size allows, by RSV-specific coded diagnoses. The three regional post-pandemic peaks now cluster within a 2-month range so that a region-tailored schedule would differ from a single national window by at most 1 month per region; because the post-pandemic estimates that would justify a region-tailored schedule are markedly less stable in Sierra and Amazonía than in Costa, we propose a single national administration window (nirsevimab February–April; maternal RSVpreF targeted to pregnancies expected to deliver January–May, ahead of the May population-weighted national peak) as the more defensible starting point for Ecuadorian RSV immunoprophylaxis policy. This recommendation rests on that instability and on administrative simplicity, not on the national window's coverage under an author-chosen tolerance, which is not itself evidence of superiority; regional and altitudinal heterogeneity is retained as a secondary finding warranting continued surveillance rather than an immediately implementable region-tailored schedule. Confirmation from additional post-pandemic seasons, and ideally from laboratory-confirmed RSV surveillance, particularly in Amazonía, would be required before a more granular, region- or altitude-specific policy could be recommended with confidence.

## Data Availability

Publicly available datasets were analyzed in this study. This data can be found here: https://www.ecuadorencifras.gob.ec/camas-y-egresos-hospitalarios-informacion-historica/.
