# Supplementary material for: Regional, altitudinal, and age-specific heterogeneity of bronchiolitis hospitalization seasonality in Ecuador, 2007–2024, and implications for RSV immunoprophylaxis timing
Source: Front Public Health. 2026 Aug 3;14:1892098. doi: 10.3389/fpubh.2026.1892098 (PMC13478267; doi:10.3389/fpubh.2026.1892098)
Supplement: Supplementary file 1 [file Data_Sheet_1.pdf]

frontiers

Supplementary Material: Regional, altitudinal and age-specific heterogeneity of bronchiolitis hospitalisation seasonality in Ecuador, 2007–2024, and implications for RSV immunoprophylaxis timing

Jaime Angamarca-Iguago [1,3,*], Jaen Cagua-Ordóñez [1], Juan Marcos Parise-Vasco [1], Doménica De Mora [4], Monica Escobar Naranjo [5], Alfredo Bruno [4,6], Daniel Simancas-Racines [2,*]

[1] *Facultad de Ciencias de la Salud y Bienestar Humano, Universidad Tecnológica Indoamérica, Quito 170103, Ecuador*
[2] *Facultad de Ciencias de la Salud y Bienestar Humano, Universidad Tecnológica Indoamérica, Ambato, Ecuador*
[3] *Training Programs in Epidemiology and Public Health Interventions Network (TEPHINET), Quito, Ecuador*
[4] *National Reference Laboratory for Influenza and Other Respiratory Viruses, Instituto Nacional de Investigación en Salud Pública (INSPI), Guayaquil, Ecuador*
[5] *Dirección Nacional de Vigilancia Epidemiológica, Ministerio de Salud Pública del Ecuador, Quito, Ecuador*
[6] *Universidad Agraria del Ecuador, Guayaquil, Ecuador*

Correspondence*:
Jaime Angamarca-Iguago
jaime.angamarca@gmail.com

This Supplementary Material reports full results for three complementary and sensitivity analyses summarised in Section 3.5 of the main text: the Moving Epidemics Method (Table S1), Bai–Perron structural-break detection (Table S2), and lagged climate correlations (Table S3). These analyses are complementary or sensitivity checks rather than independent primary inference; the primary seasonality estimates are the circular metrics reported in Tables 1–4 of the main text.

S1. MOVING EPIDEMICS METHOD (MEM)

The Moving Epidemics Method (Vega et al., *Influenza Other Respir Viruses* 2015;9(5):234–46; cited in full in the main manuscript's reference list) was fitted to the monthly J21 (<12 months) series for each region and period using the R package `mem` (version 2.18), with geometric-mean thresholds, a significance level of 0.95, and intensity thresholds at the 40th, 90th and 97.5th percentiles. Table S1 reports the epidemic threshold and convergence status for each region-period stratum.

Region	Period	Epidemic threshold	Convergence status
Costa	Pre-COVID	Not estimable	Algorithm ran but did not return a stable threshold
Costa	Post-COVID	14.18	Converged
Sierra	Pre-COVID	Not estimable	MEM convergence failed
Sierra	Post-COVID	Not estimable	Algorithm ran but did not return a stable threshold
Amazonía	Pre-COVID	2.0 – 5.41 (range across threshold percentiles)	Converged
Amazonía	Post-COVID	Not estimable	MEM convergence failed

Interpretation. MEM provided a convergent post-pandemic operational threshold in Costa, but convergence was incomplete in four of the remaining five region-period strata (Costa pre-pandemic, both Sierra periods, and Amazonía post-pandemic), consistent with the year-by-year instability documented in Section 3.4 of the main text. Because convergence was incomplete across strata, these outputs are reported only as an operational cross-check on the primary circular-metric findings, not as an independent inferential framework.

S2. BAI–PERRON STRUCTURAL-BREAK DETECTION

Bai–Perron multiple-structural-break models (`rate ~ 1`) were fitted to the monthly J21 hospitalisation rate for each region, selecting the number of breakpoints m by minimum BIC (candidate models with $h = 0.15$, i.e. a minimum segment length of approximately 15 % of the 216-month series). As specified in the main text (Section 2.5.7), this model tests for a shift in the mean *level* of the monthly rate; it does not model, and cannot directly test, a shift in the within-year *timing* (phase) of the seasonal peak.

Region	m (optimal)	BIC	Breakpoint dates
Costa	3	2,001	January 2011; August 2019; April 2022
Sierra	4	2,040	December 2011; November 2015; June 2019; March 2022
Amazonía	2	1,845	November 2014; June 2018
Amazonía (candidate, not preferred)	4	1,857	Includes a March 2022 breakpoint, but has higher BIC than the 2-breakpoint model above and is therefore not the statistically preferred partition

Interpretation. The optimal partitions place breakpoints in Costa and Sierra close to the 2022 school-reopening/NPI-withdrawal period, which we interpret as corroborating, but not validating, a concurrent change in seasonal phase (the phase shift itself is characterised by the circular metrics in Section 3.2 of the main text). In Amazonía, the BIC-optimal partition does *not* select a 2022 breakpoint, reinforcing that the Amazonía post-pandemic finding should be treated as provisional.

S3. LAGGED CLIMATE CORRELATIONS (FULL RESULTS)

Table S3 reports, for all $3 \times 4 \times 4 = 48$ region-by-exposure-by-lag combinations, the nominal Pearson correlation, nominal p-value, autocorrelation-adjusted effective sample size (N_{eff}, via the Bretherton et al. 1999 formula), the autocorrelation-adjusted p-value, and the Benjamini–Hochberg FDR-adjusted p-value (adjusted across all 48 tests). “Survives both corrections” (bold “Yes”) indicates $p_{\text{FDR,adj}} \leq 0.05$.

Region	Exposure	Lag	r	Nominal p	N_{eff}	p_{adj}	$p_{\text{FDR,adj}}$	Survives both
Costa	Temperature	0	-0.39	0	71.9	7e-04	0.0056	**Yes**
		1	-0.312	0	71.6	0.0079	0.0345	**Yes**
		2	-0.142	0.0386	71.3	0.2381	0.4396	No
		3	0.11	0.1089	70.7	0.3614	0.5596	No
Costa	Precipitation	0	0.302	0	68.3	0.012	0.0458	**Yes**
		1	0.478	0	68.1	0	0	**Yes**
		2	0.516	0	67.9	0	0	**Yes**
		3	0.378	0	67.5	0.0016	0.0096	**Yes**
Costa	NDVI	0	0.504	0	54.5	1e-04	0.0012	**Yes**
		1	0.507	0	54.5	1e-04	0.0012	**Yes**
		2	0.361	0	54.3	0.0072	0.0345	**Yes**
		3	0.086	0.2102	53.8	0.5361	0.6433	No
Costa	Niño 1+2 anomaly	0	0.188	0.0056	47.2	0.2051	0.4102	No

Region	Exposure	Lag	r	Nominal p	N_{eff}	p_{adj}	$p_{\mathrm{FDR,adj}}$	Survives both
		1	0.128	0.0619	47	0.3931	0.56	No
		2	0.087	0.204	46.7	0.5615	0.6574	No
		3	0.055	0.423	46.4	0.7144	0.762	No
Sierra	Temperature	0	-0.202	0.0028	67.8	0.0984	0.2486	No
		1	-0.083	0.2268	66.5	0.507	0.6404	No
		2	0.079	0.2469	66.5	0.524	0.6433	No
		3	0.254	2e-04	66.8	0.0383	0.1067	No
Sierra	Precipitation	0	0.259	1e-04	68.2	0.0324	0.1037	No
		1	0.254	2e-04	68	0.0364	0.1067	No
		2	0.16	0.019	67.9	0.192	0.4007	No
		3	-0.033	0.6326	68.2	0.7894	0.8062	No
Sierra	NDVI	0	0.367	0	45.7	0.0124	0.0458	**Yes**
		1	0.24	4e-04	45.7	0.109	0.2616	No
		2	0.047	0.4899	46	0.7543	0.7871	No
		3	-0.169	0.0135	46.4	0.2592	0.4536	No
Sierra	Niño 1+2 anomaly	0	0.179	0.0082	35.3	0.3	0.493	No
		1	0.151	0.0269	35.2	0.3853	0.56	No
		2	0.148	0.0306	35	0.3967	0.56	No
		3	0.13	0.0575	35.1	0.4549	0.5926	No
Amazonía	Temperature	0	-0.046	0.5034	125.2	0.6119	0.6831	No
		1	0.101	0.1411	124.6	0.2646	0.4536	No
		2	0.185	0.0067	124	0.04	0.1067	No
		3	0.221	0.0012	123.6	0.0137	0.047	**Yes**
Amazonía	Precipitation	0	0.259	1e-04	147	0.0016	0.0096	**Yes**
		1	0.223	0.001	146.1	0.0068	0.0345	**Yes**
		2	0.113	0.0985	145.7	0.174	0.3977	No
		3	-0.012	0.8675	145.8	0.8905	0.8905	No
Amazonía	NDVI	0	-0.066	0.3358	132.1	0.4533	0.5926	No
		1	0.038	0.5835	131.9	0.6687	0.7295	No
		2	0.115	0.0936	131.2	0.1909	0.4007	No
		3	0.318	0	130.3	2e-04	0.0019	**Yes**
Amazonía	Niño 1+2 anomaly	0	0.079	0.246	90.3	0.4568	0.5926	No
		1	0.057	0.4076	90	0.5952	0.6802	No
		2	0.109	0.1114	89.2	0.3081	0.493	No
		3	0.133	0.0527	88.2	0.2164	0.4155	No

Interpretation. Costa precipitation is the only exposure–region combination that survives both corrections at all four lags. Costa temperature (lags 0–1) and NDVI (lags 0–2), Sierra NDVI (lag 0), and, more modestly, Amazonía precipitation (lags 0–1), temperature (lag 3) and NDVI (lag 3) survive at a subset of lags. ENSO Niño 1+2 anomalies do not survive correction in any region at any lag. Because both the climate exposures and the hospitalisation rate share a strong annual seasonal cycle that the lag-1 autocorrelation adjustment does not fully remove, even the surviving correlations should be interpreted as robust *associations* rather than as evidence that has fully excluded shared seasonality as an explanation (main text, Section 3.5 and Limitations).
